# Retrospective application of transposon-directed insertion-site sequencing to investigate niche-specific virulence of *Salmonella* Typhimurium in cattle

**DOI:** 10.1186/s12864-018-5319-0

**Published:** 2019-01-08

**Authors:** Prerna Vohra, Roy R. Chaudhuri, Matthew Mayho, Christina Vrettou, Cosmin Chintoan-Uta, Nicholas R. Thomson, Jayne C. Hope, John Hopkins, Mark P. Stevens

**Affiliations:** 1 The Roslin Institute and Royal (Dick) School of Veterinary Studies, University of Edinburgh, Easter Bush, Edinburgh, EH25 9RG UK; 2 Department of Molecular Biology and Biotechnology, University of Sheffield, S10 2TN, Sheffield, UK; 3 Wellcome Trust Sanger Institute, Hinxton, Cambridge, CB10 1SA UK

**Keywords:** *Salmonella*, TraDIS, Niche-specific virulence, Zoonoses

## Abstract

**Background:**

*Salmonella enterica* subspecies *enterica* is an animal and zoonotic pathogen of global importance. Cattle are a significant reservoir of human non-typhoidal salmonellosis and can suffer enteric and systemic disease owing to the ability of *Salmonella* to survive within the bovine lymphatic system and intestines. Contamination of food can occur due to the incorporation of contaminated peripheral lymph nodes or by direct contamination of carcasses with gut contents. It is essential to understand the mechanisms used by *Salmonella* to enter and persist within the bovine lymphatic system and how they differ from those required for intestinal colonization to minimize zoonotic infections.

**Results:**

Transposon-directed insertion site sequencing (TraDIS) was applied to pools of mutants recovered from mesenteric lymph nodes (MLNs) draining the distal ileum of calves after oral inoculation with a library of 8550 random *S*. Typhimurium mini-Tn*5*Km2 mutants in pools of 475 mutants per calf. A total of 8315 mutants representing 2852 different genes were detected in MLNs and their in vivo fitness was calculated. Using the same improved algorithm for analysis of transposon-flanking sequences, the identity and phenotype of mutants recovered from the distal ileal mucosa of the same calves was also defined, enabling comparison with previously published data and of mutant phenotypes across the tissues. Phenotypes observed for the majority of mutants were highly significantly correlated in the two tissues. However, 32 genes were identified in which transposon insertions consistently resulted in differential fitness in the ileal wall and MLNs, suggesting niche-specific roles for these genes in pathogenesis. Defined null mutations affecting *ptsN* and *spvC* were confirmed to result in tissue-specific phenotypes in calves, thus validating the TraDIS dataset.

**Conclusions:**

This validation of the role of thousands of *Salmonella* genes and identification of genes with niche-specific roles in a key target species will inform the design of control strategies for bovine salmonellosis and zoonotic infections, for which efficacious and cross-protective vaccines are currently lacking.

**Electronic supplementary material:**

The online version of this article (10.1186/s12864-018-5319-0) contains supplementary material, which is available to authorized users.

## Background

*Salmonella enterica* subspecies *enterica* is a bacterial pathogen of global importance for humans and animals. The World Health Organisation estimated that *S. enterica* caused 78 million cases of foodborne illness, 59,000 deaths and the loss of 4.1 million disability-adjusted life years in 2010 [1]. Farmed animals are key reservoirs of human non-typhoidal salmonellosis and infections are frequently associated with ingestion or handling of contaminated meat. In the United States, *Salmonella* is endemic in cattle and human infections have been attributed to both beef and dairy cattle [2].

*S. enterica* can survive within the bovine lymphatic system and contaminate peripheral lymph nodes [3–6]. Peripheral lymph nodes are small and embedded in adipose tissue within parts of the carcass that are incorporated into ground beef products, making it unfeasible to remove them on the scale of modern beef production. Improper cooking of contaminated ground beef can therefore result in sporadic human infections and outbreaks [7–11]. A survey of beef cattle with a high rate of *Salmonella* carriage in faeces, also detected contamination of peripheral lymph nodes but the prevalence of serovars was found to vary between niches. For example, serovar Meleagridis was more frequently recovered from lymph nodes and serovar Kentucky was more commonly recovered from faeces, suggesting differences in the ability of different serovars to enter and survive within the lymphatic system [12]. However, by whole-genome sequencing and analysis of polymorphic alleles, it was recently demonstrated that diverse serovars are capable of contaminating the bovine lymphatic system during mixed infections and thus pose similar zoonotic risks [3].

*S. enterica* causes disease in cattle as well, from acute enteritis caused by serovar Typhimurium to severe systemic typhoid-like disease caused by serovar Dublin, which can also lead to abortion [13]. Bovine salmonellosis can be fatal, and systemic disease, even when treated, can result in a long-term carrier state [13]. Thus, *Salmonella* not only poses a risk to human food safety and health but is also a threat to animal welfare and productivity.

Vaccines and interventions that effectively control *Salmonella* infections in cattle are currently lacking. To address this, libraries of transposon-insertion mutants of *S*. Typhimurium have been screened in calves to assign roles to *Salmonella* genes in intestinal colonization [14–17]. The most comprehensive of these studies used transposon-directed insertion-site sequencing (TraDIS), which relies on massively-parallel sequencing of transposon-flanking regions to simultaneously identify the insertion-site of each transposon and quantify the abundance of the cognate mutant in complex pools from the number of site-sequence reads of each transposon insertion [18]. By comparing the abundance of mutants in pools before and after screening in animals, it is possible to calculate a fitness score for each mutant. By oral inoculation of calves, 8550 miniTn*5*Km2 mutants of *S*. Typhimurium ST4/74 were screened in pools of 475 per calf and roles were assigned to 2715 genes in intestinal colonization [15]. For systemic infection, however, it is essential to understand the mechanisms that enable *Salmonella* to translocate from the intestines into the lymphatic system and disseminate within it.

The translocation of *Salmonella* from the intestinal mucosa to mesenteric lymph nodes (MLNs) is best characterised in mice, where migratory monocytes and granulocytes play an important role (reviewed in [19]) and MLNs confine infected migratory dendritic cells to limit systemic spread of the bacteria [20]. In cattle, the correlation of translocation with host-specificity was demonstrated using a surgical cannulation model: systemic disease-causing *S*. Dublin translocated in large numbers in the efferent lymph, while avirulent *S*. Gallinarum was less proficient at translocation despite being as invasive as *S*. Dublin in the gut [21, 22]. Translocation is relevant to bacterial dissemination during systemic salmonellosis as confirmed by the detection of identical populations of signature-tagged *S*. Dublin mutants in livers, spleens and efferent lymph of orally-infected cattle [22–24]. While early entry into the bovine lymphatic system did not depend on *Salmonella* pathogenicity island (SPI)-2, it strongly depended on forced uptake mediated by SPI-1 [22]. SPI-2 mutants could spread to bovine MLNs and efferent lymph, in contrast to observations in mice where SPI-2 has been implicated in traversal of epithelial cells and basolateral exit prior to uptake by lamina propria phagocytes [25] and triggering migration of infected phagocytes [26, 27].

While TraDIS has enhanced our understanding of the *Salmonella* genes required to colonize the bovine intestines [15], the bacterial mechanisms involved in persistence within the bovine lymphatic system are relatively poorly understood. Studies with signature-tagged mutants lacking predicted sensor kinases of two-component sensory systems have indicated that niche-specific phenotypes can occur depending on whether mutants are delivered orally or intravenously [24]. In this study, TraDIS was applied retrospectively to bacterial mutant populations recovered from MLNs archived from the calves used in the study by Chaudhuri et al., 2013. Phenotypes of mutants isolated from the intestinal mucosa and MLNs were compared and genes with putative niche-specific roles in virulence were identified.

## Results

### Assignment of phenotypes to *S*. Typhimurium mutants in the ileal wall and mesenteric lymph nodes of cattle

TraDIS was applied to *S*. Typhimurium populations recovered from the ileal wall and MLNs of calves that were orally challenged with a library of 8550 mini-Tn*5*Km2 mutants of strain ST4/74 *nal*^*R*^ at 475 mutants per calf [15] (Additional file 1: Figure S1). A total of 8315 distinct transposon insertions were mapped to the *S*. Typhimurium genome (Additional file 2: Table S1). Of these insertions, 7333 were in genes and 982 were in intergenic regions. Transposon insertions were identified in a total of 2852 genes and 1673 of these genes had more than one insertion. Transposon insertions were randomly distributed around the chromosome and plasmids of *S*. Typhimurium and there was no evidence of stochastic loss of mutants in output pools recovered from the ileal wall and MLNs of infected calves (Additional file 3: Figure S2).

A fitness score was assigned to each insertion, which is defined as the log_2_-fold change in the number of sequence reads obtained across the boundaries of the transposon insertion between the input and output pools. Because of improvements in transposon identification and analysis by *DESeq2*, a newer version of *DESeq*, which was used to analyse our previous TraDIS dataset [15], fitness scores were assigned to an additional 1478 transposon insertions. Of these, 1289 insertions were in 954 genes (Additional file 4: Table S2). Moreover, 1145 insertions, which were previously assigned an arbitrary value of − 15 owing to the lack of reads in output pools, were assigned fitness scores based on the distribution of scores for genes with non-zero output read counts using *DESeq2* [15]. A user-friendly online genome browser has been created using Dalliance (PMID: 21252075) to display the location and phenotype of transposon insertions (http://hactar.shef.ac.uk/SL1344).

### Identification of genes with differential fitness in the ileal wall and mesenteric lymph nodes

**Fig. 1:**
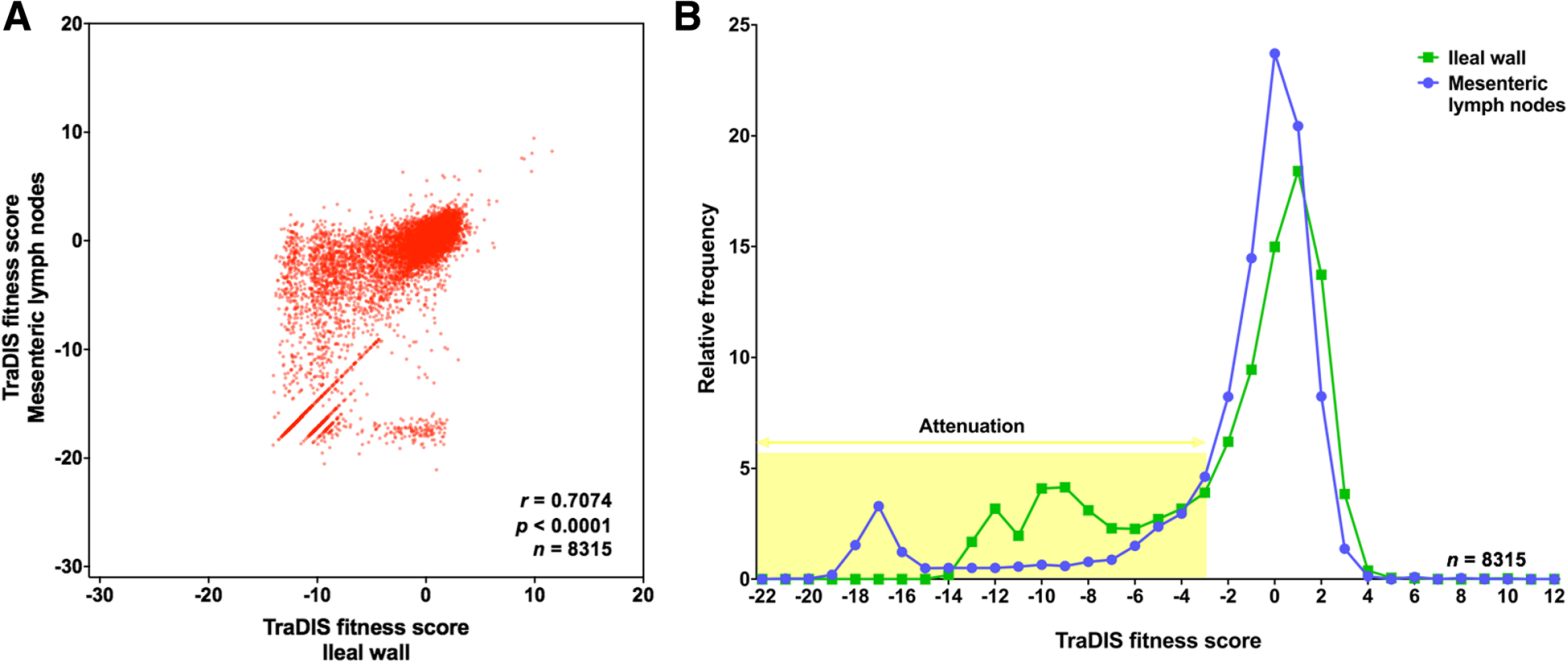
Correlation of fitness scores of *S*. Typhimurium transposon-insertion mutants in the ileal wall and mesenteric lymph nodes and distribution of fitness scores. (**a**) A correlation plot showing a high degree of correlation between fitness scores of 8315 transposon-insertion mutants identified in the ileal wall and MLNs suggests that majority of mutants have similar phenotypes in both tissues. (**b**) Histograms of the fitness scores of mutants in the ileal wall and MLNs show that a large proportion of mutants have fitness scores approximate to zero in both tissues, suggesting that many insertions have no effect on phenotype. Based on fitting of bimodal distributions of fitness scores in the histograms, a fitness score of ≤ − 3.0 was selected as the cut-off value to define attenuation (within the yellow box)

Correlation analysis of the fitness scores of transposon-insertion mutants recovered from the ileal wall and MLNs showed that over 83% of mutants had similar phenotypes in both tissues (*r* = 0.7074) (Fig. 1a). As reported previously, for a large proportion of mutants the fitness scores approximate to zero in both tissues (log_2_ = 0), indicating that those mutants were present at comparable levels in the input and output pools. This suggests that their mutations have no effect on phenotype and indicate a high level of functional redundancy in the genome of *S*. Typhimurium (Fig. 1b). From the distribution of the fitness scores of mutants in both tissues, a cut-off value of ≤ − 3.0 was selected to describe attenuation and 1445 transposon insertions were found to be attenuating in both tissues (Additional file 5: Table S3). Of these, 1289 were in genes and 156 were in intergenic regions. In 653 genes, one or more insertions were attenuating in both tissues. In 131 genes, all the insertions were attenuating in both tissues and these included SPI-1, SPI-2 and O-antigen synthesis genes, as expected. In 3 genes, *cpsB2*, *rfaL* and *sipA*, the majority of the transposon insertions were strictly attenuating by our definition; at the few positions where fitness scores were not strictly attenuating, a trend towards attenuation was observed.

**Fig. 2:**
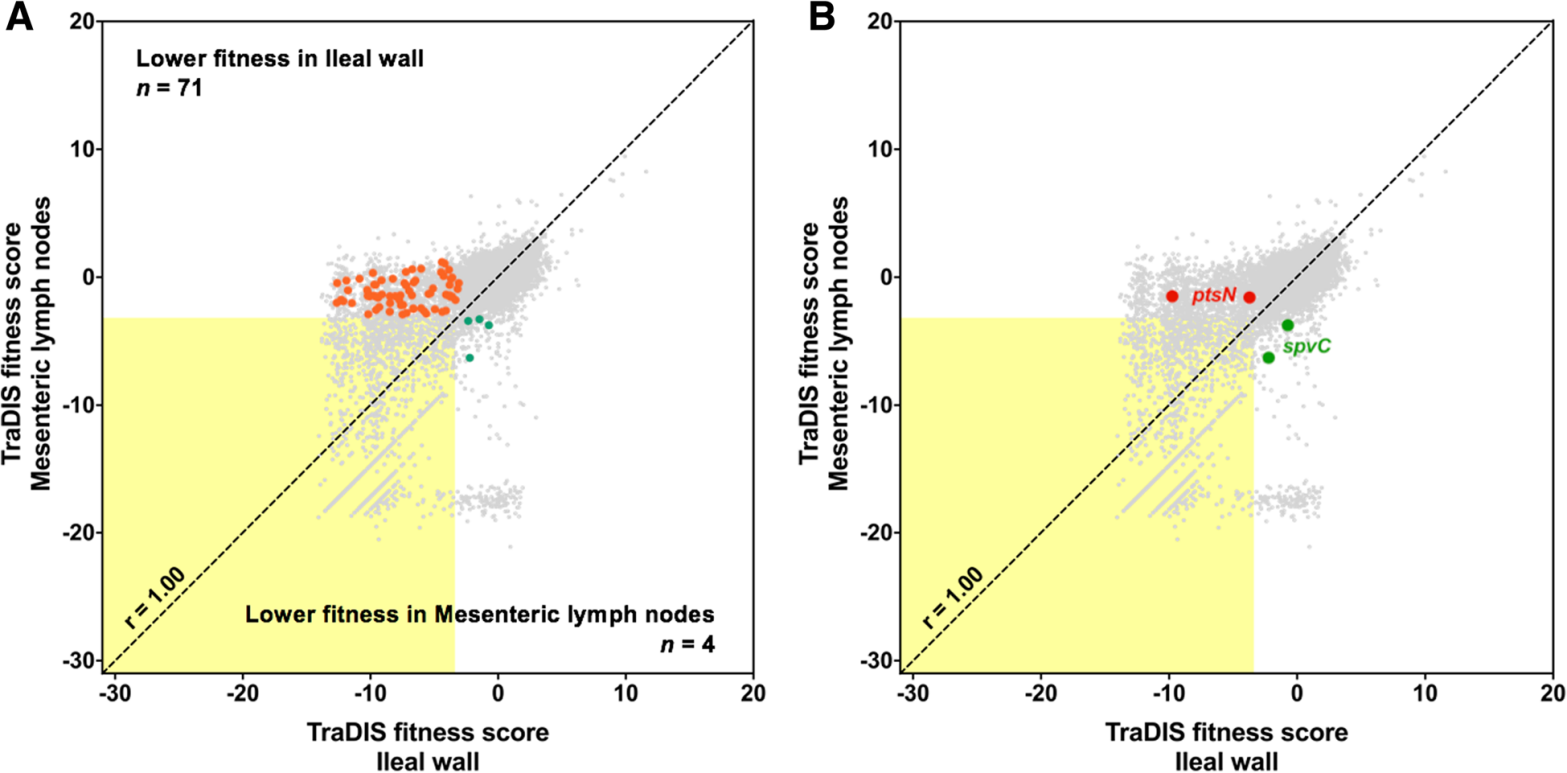
Identification of *S*. Typhimurium transposon-insertion mutants with putative niche-specific differences in fitness. (**a**) A correlation plot showing mutants with differential fitness superimposed on all mutants [] and those attenuated in both tissues (within the yellow box). Orange dots [] indicate mutants of genes which were consistently attenuated only in the ileal wall and green dots [] indicate mutants of genes which were consistently attenuated only in MLNs. (**b**) A correlation plot highlighting the mutants of the 2 genes selected for validation: *ptsN* [] and *spvC* []

To identify genes with differential fitness in the ileal wall and MLNs, firstly, insertions which were attenuating in only one of the two tissues were identified. Accordingly, 1100 insertions in 951 genes were attenuating in the ileal wall only and 294 insertions in 257 genes were attenuating in MLNs only (Additional file 5: Table S3). Genes with a single insertion and genes in which insertions at different positions resulted in dissimilar fitness scores, possibly owing to varying effects on the encoded protein, were excluded. From the remaining genes, those which had the same phenotypes in more than one animal, were selected. Thirty genes had lower fitness in the ileal wall and 2 genes had lower fitness in MLNs (Fig. 2a). To account for the possibility that insertions could result in differential fitness without causing strict attenuation at every insertion site, genes in which all insertions resulted in reduced fitness in only 1 tissue, and at least one of these had a fitness score of ≤ − 3, were shortlisted: 85 and 24 genes with lower fitness in the ileal wall and MLNs, respectively, were identified (Additional file 5: Table S3).

### Validation of selected TraDIS findings using defined null mutants

**Fig. 3:**
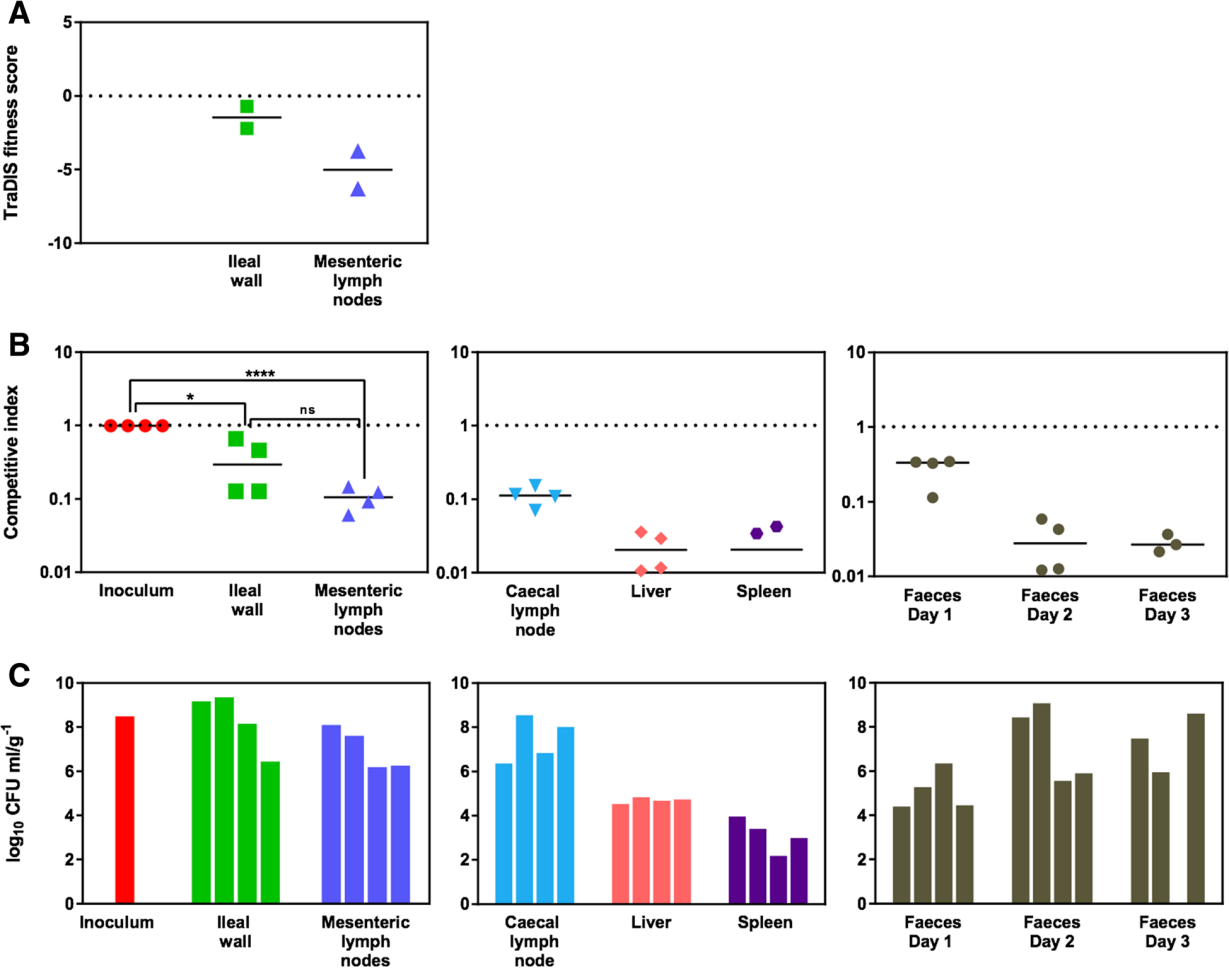
In vivo validation of the phenotype of a *S*. Typhimurium *ptsN* mutant. (**a**) The TraDIS fitness scores of transposon-insertion mutants of *ptsN* indicate attenuation in MLNs but not in the ileal wall. (**b**) Competitive index scores of ST4/74 *nal*^*R*^ ∆*ptsN*::*cat* compared to the wild-type ST4/74 *nal*^*R*^ confirmed the phenotype predicted by TraDIS in the ileal wall and MLNs. CIs in the caecal lymph node, liver and spleen show attenuation of ST4/74 *nal*^*R*^ ∆*ptsN*::*cat* in these tissues. CIs in faecal samples decreased over time. (**c**) Bacterial counts confirmed the total challenge dose of 3.07 × 10^8^ CFU of mutant and wild-type bacteria and recovery of viable bacteria per tissue per calf is shown

**Fig. 4:**
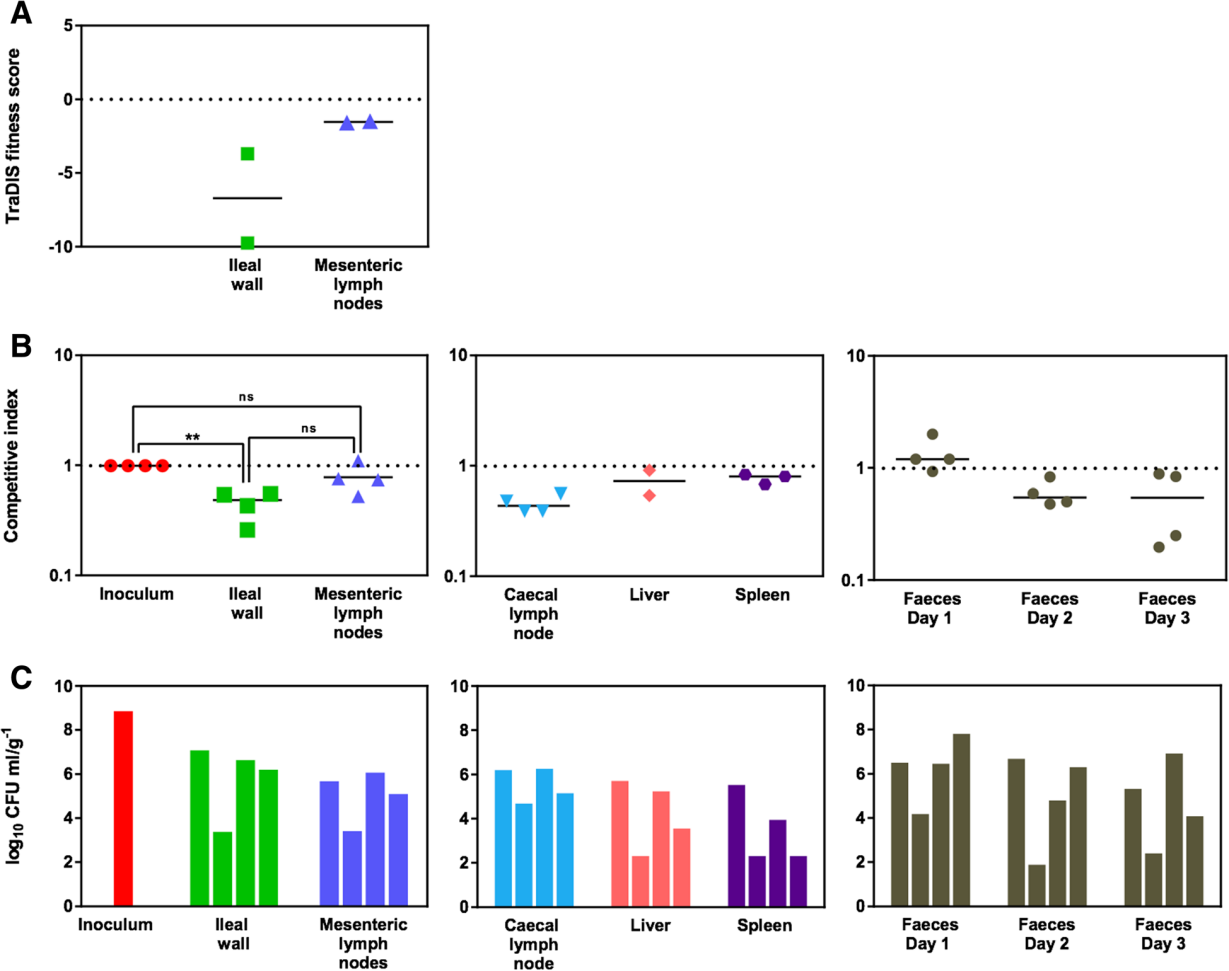
In vivo validation of the phenotype of a *S*. Typhimurium *spvC* mutant. (**a**) The TraDIS fitness scores of transposon-insertion mutatns of *spvC* indicate attenuation in the ileal wall but not in MLNs. (**b**) Competitive index scores of ST4/74 *nal*^*R*^ ∆*spvC*::*cat* compared to the wild-type ST4/74 *nal*^*R*^ confirmed the phenotype predicted by TraDIS in the ileal wall and MLNs. CIs in the caecal lymph node were similar to those in the ileal wall but CIs in the liver and spleen show no attenuation of ST4/74 *nal*^*R*^ ∆*spvC*::*cat* in these tissues. CIs in faecal samples decreased over time. (**c**) Bacterial counts confirmed the total challenge dose of 7.2 × 10^8^ CFU of mutant and wild-type bacteria and recovery of viable bacteria per tissue per calf is shown

To confirm that the niche-specific differential fitness of mutants predicted by TraDIS reflects their phenotypes in vivo, two genes were selected for validation: *ptsN*, which had a lower fitness score in MLNs than in the ileal wall, and *spvC*, which had a lower fitness score in the ileal wall than in MLNs (Fig. 2b). Whole-genome sequencing of the mutants confirmed that only the expected mutations were present relative to the published ST4/74 genome (49; data not shown). Mutant phenotypes were evaluated by co-infection of 4 calves per mutant at a 1:1 ratio with the isogenic wild-type strain and a competitive index (CI) was derived (Additional file 6: Figure S3). The CIs obtained for ST4/74 *nal*^*R*^ Δ*ptsN*::*cat* in the ileal wall were higher than those in MLNs (Fig. 3), whereas the CIs for ST4/74 *nal*^*R*^ Δ*spvC*::*cat* showed the opposite trend (Fig. 4). Although statistical significance between CIs of mutants in the ileal wall and MLNs was not obtained, attenuation of ST4/74 *nal*^*R*^ Δ*ptsN*::*cat* (*P* < 0.05) and ST4/74 *nal*^*R*^ Δ*spvC*::*cat* (*P* < 0.01) was observed relative to the inoculum. In MLNs, ST4/74 *nal*^*R*^ Δ*ptsN*::*cat* was further attenuated relative to the inoculum (*P* < 0.0001), while ST4/74 *nal*^*R*^ Δ*spvC*::*cat* was not. Thus, the direction of selection for both mutants was as predicted by TraDIS. The CIs obtained in the caecal lymph node (CLN), liver and spleen for ST4/74 *nal*^*R*^ Δ*ptsN*::*cat* were also lower than those in the ileal wall, suggesting that *ptsN* may play a role in dissemination. For ST4/74 *nal*^*R*^ Δ*spvC*::*cat*, the CIs in the CLN were comparable to those in the ileal wall but the CIs in the liver and spleen reflected the phenotype of this mutant in MLNs, suggesting that it might be more important for intestinal colonization than systemic spread. The CIs of both mutants decreased in the faeces after 2 days post-inoculation, irrespective of the CI in the ileal wall.

## Discussion

Retrospective application of TraDIS to archived tissues assigned phenotypes to 2852 genes of *S*. Typhimurium in colonization of the intestines and draining mesenteric lymph nodes in cattle, a naturally affected and relevant host, following inoculation by the natural route of exposure. The robustness of the TraDIS data was demonstrated by the high degree of correlation of phenotypes of mutants in the ileal wall and MLNs and independent validation of two null mutants, which exhibited differential fitness in the two niches during the in vivo TraDIS screen. Moreover, observations pertaining to phenotypes of genes with well-defined roles in virulence and a high level of functional redundancy in the *S*. Typhimurium genome were independently replicated in this study, lending further confidence in the data.

Of the 8550 *S*. Typhimurium transposon-insertion mutants previously screened in pools of 475 in 18 calves, 8315 transposon insertions were detected in the screened inocula, which were also detected in the bacterial mutant populations recovered from both the ileal wall and MLNs. The high degree of correlation of fitness scores of mutants in both tissues suggests that most genes involved in intestinal colonization are also required for survival within lymph nodes. Multiple mutations in the same pathway also had the same effect on fitness in both tissues, thus, self-validating the data and confirming that the mutant phenotypes observed were unlikely to be a consequence of chance events. As expected, mutations affecting SPI-1, SPI-2 and O-antigen biosynthesis genes were highly attenuating in both tissues [22] (Additional file 5: Table S3). Thus, preventing colonization of the intestines of cattle can be predicted confidently to reduce the entry and persistence of *Salmonella* in the bovine lymphatic system, and minimize the food safety risk of ground beef. Occasionally, as previously described [15], transposon insertions were identified in essential genes such as RNA polymerase genes *rpoB* and *rpoC* and genes involved in translation such as *rpsA*, *rpsK* and *fusA*. However, these were single insertions at the extreme 3′ end of the genes that are predicted to result in only a minor C-terminal truncation that may not ablate protein function. Also, not all transposon insertions in a given gene resulted in the same fitness in vivo, most likely due to different effects on the cognate protein, such as causing truncations at different positions. As different mutants may have been screened in different pools, it is also possible that this variability in fitness was a result of different competition dynamics within the pools.

An important consideration in screens of this type is the potential for stochastic loss of mutants for reasons unrelated to their genotype. This can occur owing to sample size, where insufficient colonies are sampled to be confident that a mutant is absent owing to its genotype as opposed to chance. However, typically in excess of 5 × 10^5^ bacteria were recovered per MLN, at which level one can be > 95% confident that absence of mutants is due to their genotype [28]. Another cause of stochastic loss can be ‘bottleneck’ effects, whereby only a subset of the mixed population will be able to establish infection, as reported before using wild-type isogenic tagged clones screened at varying inoculum sizes in mice [21, 22]. No obvious stochastic loss of mutants from MLNs was evident in our screen; almost all of the mutants presented in the ileal wall were also resident in MLNs. Mutants absent in this screen are therefore likely to be genuinely attenuated in cattle. The absence of bottleneck effects in migration of mutants from the ileal wall to draining lymph nodes and beyond, to efferent lymph and visceral organs, was also evident from screening signature-tagged mutants of *S*. Dublin in calves [22, 23, 29], albeit at lower pool complexities than used here.

A major advantage of this study was the comparison of phenotypes of transposon-insertion mutants in two tissues in vivo simultaneously. Genes with multiple transposon insertions, which resulted in consistently differential fitness scores in the ileal wall and MLNs, with the same phenotypes observed in more than one animal were shortlisted as genes with potential niche-specific roles in virulence. Of the 32 genes identified, two were validated by co-infection studies and derivation of competitive indices: *ptsN*, which was more attenuated in MLNs than the ileal wall, and *spvC*, which showed the inverse phenotype. Of the remaining 30 genes with predicted differential fitness by TraDIS, some have been described to play roles in nutrient metabolism and sensing environmental signals, in *Salmonella* or other bacterial species (Additional file 5: Table S3).

In vivo, *ptsN* has been shown to be important for virulence in acute and chronic *S.* Typhimurium infections in mouse models with *ptsN* mutants either being recovered in fewer numbers from systemic sites such as the liver and spleen or not at all during the later stages of infection [30, 31]. EIIA^Ntr^, encoded by *ptsN*, directly interacts with SsrB thereby controlling the timing and levels of expression of SPI-2 genes and manipulating host responses to aid bacterial survival in vivo [30]. It has been observed that following uptake by murine macrophages, *ptsN* mutants do not actively replicate but persist within them [30]. SPI-2 mutants of *S. enterica* have impaired survival at enteric and systemic sites in mice and cattle during late infection, generally beyond 12 h [14, 22, 32–34]. Therefore, it is likely that the reduced fitness of *ptsN* transposon-insertion mutants in MLNs is consistent with a role in regulating SPI-2. Moreover, the *ptsN* null mutant was also attenuated in the CLN, liver and spleen of infected calves, consistent with previous observations in mice.

EIIA^Ntr^ also directly interacts with GlmS, a key enzyme in the biosynthesis of amino sugars that make up peptidoglycan and LPS of bacteria, which are important for bacterial survival and virulence [35]. Further, it has been proposed that EIIA^Ntr^ may play a role in modulating virulence of *S. enterica* by enabling it to utilize nutrients in the host gut such as 1,2-propanediol and propionate, which in turn serve as signals to upregulate the expression of SPI-1 and SPI-4 genes and enhance the invasiveness of *S. enterica* [36]. However, attenuation of *ptsN* mutants was not observed by TraDIS or competitive index in the ileum of calves.

The *spv* operon of *S. enterica* is expressed under stresses such as heat shock and glucose starvation [37, 38] and has been shown to play a role in systemic infections in mice [33, 38–40]. In cattle, however, it has been reported that the *spv* genes are not essential for enteritis or systemic spread but are important for persistence within organs [41, 42]. SpvC, encoded by this locus, is a SPI-1 and SPI-2 effector [43] and *spvC* is expressed at greater levels under nutrient-limiting conditions [44]. *spvC* was also found to be essential for the expression and function of other genes within the operon [37, 39]. It was observed that during murine intra-peritoneal infection, an *spvC* mutant was attenuated, with fewer bacteria detected in systemic organs [43, 44]. However, in orally-challenged mice, similar numbers of mutant and wild-type bacteria were recovered from spleens, suggesting that *spvC* does not affect survival within organs [43]. A similar observation was made in this study; *spvC* appears to have little effect on survival within bovine MLNs, livers and spleens.

In vitro and in vivo, SpvC has been found to reduce the levels of pro-inflammatory cytokines produced by host cells [43–45], suggesting that *spvC* may play a role in bacterial dissemination. These effects were either observed during the early stages of infection [43] or at later stages when colitis was induced by the SPI-2 alternative pathway [46]. Dissemination of *Salmonella* from the gut in cattle occurs rapidly after infection, as observed by surgical cannulation of lymph vessels [22]. In this study, a mutant of *spvC* was attenuated in the ileal wall but not in MLNs, suggesting that it may indeed play a role in dissemination. It also possible that, much like SPI-2 genes, *spvC* helps to maintain an intestinal population of bacteria for systemic dissemination, rather than being vital for translocation per se.

While TraDIS allowed an extremely comprehensive functional analysis of the *S*. Typhimurium genome, it should be noted that the predicted phenotypes of mutants can differ from published observations. For example, SPI-5 genes *pipB2*, *pipC* and *pipD* were attenuated in both tissues by TraDIS in the present study, but were not attenuated in a bovine ileal loop model using a TraDIS-like method. In such a model, however, bacteria bypass the natural host defences which would be encountered during infection by the natural oral route. Bacterial pools were also recovered earlier after inoculation, which may partly account for some of the disparities [14, 42]. For example, *yfeJ* and *sciS* were identified as genes under selection in the intestine at 12 h post-inoculation [16] but these phenotypes were not observed by TraDIS at 4 days post-infection by oral challenge. However, a SPI-3 mutant of *mgtC* showed similar phenotypes in the ileal wall and lymph nodes by TraDIS, as previously observed, providing some correlation with published observations [22]. As salmonellosis caused by *S*. Typhimurium in cattle closely resembles that in humans, many of the predicted virulence determinants are likely to be relevant to human disease, as previously proposed [42].

## Conclusions

TraDIS has been used previously for a functional analysis of the *S. enterica* genome under different conditions [15, 18, 47] and in this study, it has been used to identify genes with putative niche-specific roles that allow *S*. Typhimurium to colonize the lymphatic system as well as the intestines of cattle with minimal additional animal use. Survival of *Salmonella* within the bovine lymphatic system has direct relevance to its control in cattle and its entry into the food chain. Further studies will be required to elucidate the precise mechanisms by which these genes mediate niche-specific survival, and to exploit the data for the development of vaccines and other interventions.

## Materials and methods

### Oral challenge of calves with a mutant library of *S*. Typhimurium

The methods used to screen a library of *S*. Typhimurium ST4/74 *nal*^*R*^ mutants in calves by TraDIS were described previously [15]. Briefly, 8550 mini-Tn*5*Km2 mutants were generated in a spontaneous nalidixic acid resistant variant of *S*. Typhimurium ST4/74 [48]. Eighteen 25- to 32-day-old Friesian bull calves were orally challenged with separate pools containing 475 mutants. Animal experiments were conducted at the Institute for Animal Health according to the requirements of the Animals (Scientific Procedures) Act 1986 under license 30/2485 with the approval of the local Ethical Review Committee. Calves were killed humanely at 4 days post-infection, except 1 calf that reached the clinical end-point at 3 days post-infection.

### Sample collection and processing

A section of distal ileum and draining MLNs were collected from all calves. MLNs were collected aseptically prior to rupture of the intestines and archived at − 80 °C. A 1 g full thickness biopsy of the distal ileum was homogenised in 9 ml of PBS. Serial dilutions were plated on MacConkey agar containing 20 μg ml^− 1^ nalidixic acid and 50 μg ml^− 1^ kanamycin to determine viable counts and the remaining homogenate was spread onto 20 plates to recover output pools. At least 10^5^ colonies were collected in phosphate-buffered saline (PBS) and pellets were stored at − 20 °C for DNA extraction. Archived MLNs were trimmed of excess fat and fascia and 1 g of tissue was homogenised in 9 ml of PBS using a gentleMACS Dissociator in gentleMACS M Tubes. Serial dilutions and recovery of output pools were done as before. An average of 5 × 10^5^ colonies were collected in PBS and pellets were stored at − 20 °C for DNA extraction.

Genomic DNA (gDNA) was extracted from the pellets using the NucleoSpin® Tissue kit (Macherey-Nagel) according to the manufacturer’s instructions. DNA quality and quantity were assessed by agarose gel electrophoresis and Nanodrop 3300 (Thermo Scientific). Samples with an A_260/280_ of ≥1.8 were considered suitable for library preparation.

### TraDIS analysis

gDNA was prepared from the inocula and output pools from the ileal wall and MLN of each calf, and fragmented to approximately 300 bp as described previously [15, 49]. An Illumina adapter specific to each pool was ligated to the fragments, and PCRs were performed using an adapter-specific primer in conjunction with outward-facing primers that anneal to each end of the transposon. The quantities of each amplicon were standardised by quantitative PCR. Amplicons were sequenced on single-end Illumina flowcells using a primer designed to read a 10 bp tag of transposon-derived sequence, plus 27 bp of flanking gDNA. TraDIS sequence data are available from the European Nucleotide Archive (study accession number PRJEB10723).

Sequences containing the tag were identified using Cutadapt (Ref: http://journal.embnet.org/index.php/embnetjournal/article/view/200), and mapped using BWA mem (Ref: https://arxiv.org/abs/1303.3997) to the *S*. Typhimurium SL1344 genome sequence (GenBank accession numbers FQ312003 and HE654724–6), which differs from that of ST4/74 by just 8 single nucleotide polymorphisms [50]. A transposon was inferred to be present if corresponding reads derived from each end of the transposon were identified in the input pool. Identification of transposon insertions was as described in Chaudhuri et al., 2013 with a slight modification. In the previous study, the presence of an insertion was only inferred if in the TraDIS sequence data for the input pool there were reads which mapped to opposite strands separated by 9 bp, the expected size of the duplication associated with Tn5 integration [51]. In this study, this requirement was relaxed to include reads separated by at least 8 bp and no more than 10 bp, to account for the possibility of single base insertion or deletion errors in the reads or the reference genome sequence, thus increasing the total number of mutants identified. Analysis for output pools from the ileal wall was done as in Chaudhuri et al., 2013 but *DESeq2* [52] was used instead of *DESeq* [53] since it offers improvements for data analysis. To enable a true comparison between the ileal wall and MLNs, data for the ileal wall (ENA study accession number PRJEB2231) were re-analysed using *DESeq2*. The ratio of the transposon read numbers between input and output pools was determined, after normalisation to account for variations in the total number of reads obtained for each sample, and expressed as log_2_ fold change, referred to as the fitness score. Fitness scores were also estimated for mutants which were not detected in output pools using *DESeq2*. For each mutant, the hypothesis that the fitness score equals zero (i.e. that the mutant was present at equivalent levels in the input and output pools) was tested using the negative binomial distribution as implemented in *DESeq2*. *DESeq2* models variance under the assumption that mutants with comparable levels of sequence coverage exhibit similar levels of dispersion, and allows for estimation of *P* values for all mutants whilst minimizing the number of biological replicates by fitting, using only those mutants for which replicate data points were available, and applying the resultant model to the data derived from all mutants.

### Identification of mutants with differential fitness

Correlation analysis of the fitness scores of transposon-insertion mutants recovered from the ileal wall and MLNs was performed. From the distribution of fitness scores obtained for all mutants in both tissues, a fitness score of ≤ − 3.0 was selected to define attenuation. Three criteria were applied to identify genes with differential fitness: (1) multiple transposon insertions, (2) which were attenuating in only one tissue (3) and observed in more than 1 calf.

### Construction of defined mutants for validation

Defined null mutants were generated for 2 genes with differential fitness. ST4/74 genes *ptsN* (chromosomal) and *spvC* (located on plasmid pSLT, accession no. HE654724) were mutated by λ red recombinase-mediated integration of linear PCR products as previously described [54]. Primers were designed to amplify the pKD3-encoded chloramphenicol (*cat*) resistance cassette, including 45-bp homology extensions from the 5′ and 3′ ends of the genes to be mutated (Additional file 7: Table S4) such that the entire coding sequence of the target gene would be replaced by the *cat* cassette after homologous recombination. PCR products were purified and electroporated into ST4/74 *nal*^*R*^ carrying pKD46 cultured in Luria-Bertani (LB) broth containing 100 μg ml^− 1^ ampicillin at 30 °C in the presence of 0.2% L-arabinose to induce the λ red recombinase. Recombinants were selected on MacConkey agar containing 20 μg ml^− 1^ nalidixic acid and 20 μg ml^− 1^ chloramphenicol and cured of pKD46 by culture at 37 °C in the absence of ampicillin. Mutations were confirmed by PCR with primers flanking the target genes (Additional file 7: Table S4).

The marked deletions were then transferred into wild-type ST4/74 *nal*^*R*^ by transduction with bacteriophage P22/HT*int* [55]. P22 phage was added to separate overnight LB broth cultures of the donor strains ST4/74 *nal*^*R*^ Δ*ptsN*::*cat* and ST4/74 *nal*^*R *^Δ*spvC*::*cat*, and incubated at 37 °C for 4 h. The cultures were incubated at room temperature for 10 min with 500 μL of chloroform and then centrifuged at 5000 g for 10 min. The supernatants containing the P22 transducing phages were added separately to broth cultures of recipient ST4/74 *nal*^*R*^ and incubated at 37 °C for 1 h. The transduction mixtures were plated onto LB agar containing 20 μg ml^− 1^ nalidixic acid and 20 μg ml^− 1^ chloramphenicol and incubated overnight at 37 °C. The resulting transductants were re-streaked onto green indicator agar to check for the presence of pseudolysogens. Mutations in non-pseudolysogenic transductants were confirmed by PCR with primers flanking the target genes as before.

gDNA was extracted from PCR-confirmed mutants as described above and whole genome Illumina sequencing was used for validation (performed by MicrobesNG, University of Birmingham, UK). Sequence reads were aligned to the ST4/74 genome [50] and are available from the European Nucleotide Archive (study accession number PRJEB27500).

### Oral infection of calves with mutant and wild-type bacteria in competition

The fitness of ST4/74 *nal*^*R*^ ∆*ptsN*::*cat* and ST4/74 *nal*^*R*^ ∆*spvC*::*cat* mutants was assessed in competition with wild-type ST4/74 *nal*^*R*^ in vivo. Bacterial cultures were prepared in LB broth containing the appropriate antibiotics incubated statically at 37 °C for 16 h. Approximate CFU ml^− 1^ were estimated from OD_600_ values and inocula were prepared such that mutant and wild-type ST4/74 *nal*^*R*^ were present in equal proportions. The numbers of mutant and wild-type bacteria in the inocula were confirmed retrospectively by plating ten-fold serial dilutions.

Four 28-day-old Friesian bull calves were used to test each mutant. Calves were confirmed to be culture-negative for *Salmonella* before inoculation by enrichment of faecal samples in Rappaport-Vassiliadis broth at 37 °C for 18 h, followed by plating on MacConkey agar at 37 °C for 24 h. Animal experiments were conducted at the Moredun Research Institute according to the requirements of the Animals (Scientific Procedures) Act 1986 under license 60/4420 with the approval of the local Ethical Review Committee. Calves were orally inoculated with 20 ml of a mixture of inocula and ant-acid (5% Mg(SiO_3_)_3_, 5% NaHCO_3_ and 5% MgO in sterile distilled water) by syringe before the morning feed. The inocula contained a total of 8.5 and 8.9 log_10_ CFU of mutant and wild-type bacteria for the ST4/74 *nal*^*R*^ ∆*ptsN*::*cat* and ST4/74 *nal*^*R*^ ∆*spvC*::*cat* trials, respectively. Calves were fed on a normal diet of milk following challenge and monitored every 12 h. Post-mortem examinations were performed at 4 days post-infection.

### Sample collection and processing following co-infection of calves

Faecal samples were collected daily from all calves. At post-mortem, sections of distal ileum, draining MLNs, CLNs, liver and spleen were collected from all calves. Instruments were changed for each site sampled and tissues from the gut were removed last to avoid cross-contamination. Samples were collected in an isotonic medium (0.75% choline chloride, 0.27% KCl, 1.8% glucose, 0.5% choline bicarbonate, 1% 10X Minimum Essential Medium Eagle with Earle′s salts, 1% foetal calf serum, 20 mM L-glutamine and 0.3% NaHCO_3_ in distilled water) and promptly transported to the laboratory on ice. Ileum and lymph nodes were processed as described before. A 1 g section from all tissues was homogenized in 9 ml of PBS. Serial dilutions were plated on MacConkey agar with either only 20 μg ml^− 1^ nalidixic acid (to enumerate total *Salmonella*) or 20 μg ml^− 1^ nalidixic acid and 20 μg ml^− 1^ chloramphenicol (to enumerate the defined mutants) and incubated overnight at 37 °C. Competitive indices (CIs) were calculated as the ratio of mutant to wild-type in output pools divided by the ratio of mutant to wild-type in the inocula. The significance of any differences was tested by ANOVA and Tukey’s multiple comparisons test in GraphPad Prism version 7.00 (GraphPad Software). *P* values ≤0.05 were considered to be significant.

## Additional files

Additional file 1: **Figure S1.** Experimental strategy to screen TraDIS mutants in vivo*.* An input pool of random transposon-insertion mutants was generated and used to orally inoculate 28-day-old calves. Output pools of bacteria were recovered from tissues of interest, here the ileal wall and MLNs. Massively-parallel sequencing of the regions flanking each transposon allowed disrupted genes to be identified. A comparison of the number of sequence reads derived from the input and output pools at each transposon insertion allowed the relative fitness of each mutant to be assessed. Modified from Chaudhuri et al., 2013. (TIFF 386 kb)

Additional file 2: **Table S1.** The complete TraDIS dataset showing the positions of all transposon insertions in the genome of *S*. Typhimurium and a list of all genes with insertions. (XLSX 1347 kb)

Additional file 3: **Figure S2.** Circular diagrams of the *S*. Typhimurium chromosome and plasmids showing the distribution and abundance of mapped transposon insertions. Transposon insertions were randomly distributed across the chromosome and plasmids of *S*. Typhimurium in the input pool and output pools from the ileal wall and MLNs. The inner two rings indicate the positions of annotated genes, coloured according to GC content (blue = low, yellow = intermediate, red = high). The outer ring indicates the number of transposon-flanking sequence reads obtained at each position. Peak heights are scaled relative to the highest peak in each diagram. (TIFF 914 kb)

Additional file 4: **Table S2.** All newly identified transposon-insertion mutants and mutants with newly resolved fitness scores. (XLSX 498 kb)

Additional file 5: **Table S3.** All genes attenuated in the ileal wall and MLNs and genes with differential fitness in both tissues. (XLSX 675 kb)

Additional file 6: **Figure S3.** Experimental strategy to validate attenuated mutants in vivo. To confirm the differential fitness of *ptsN* and *spvC* in the ileal wall and MLNs, null mutants were tested in competition with wild-type ST4/74 *nal*^*R*^ in vivo. Calves were challenged orally with an inoculum containing mutant and wild-type bacteria in equal proportions. Bacteria were recovered from faeces daily and from the distal ileum, MLNs and other tissues at 4 days post-infection by plating on selective agar and the competitive indices were determined for each tissue. (TIFF 296 kb)

Additional file 7: **Table S4.** Primers used to generate and confirm marked null mutants of *ptsN* and *spvC*. (XLSX 11 kb)
