# Supplementary figures and images for: Retrospective application of transposon-directed insertion-site sequencing to investigate niche-specific virulence of Salmonella Typhimurium in cattle
Source: BMC Genomics. 2019 Jan 8;20:20. doi: 10.1186/s12864-018-5319-0 (PMC6325888; doi:10.1186/s12864-018-5319-0)

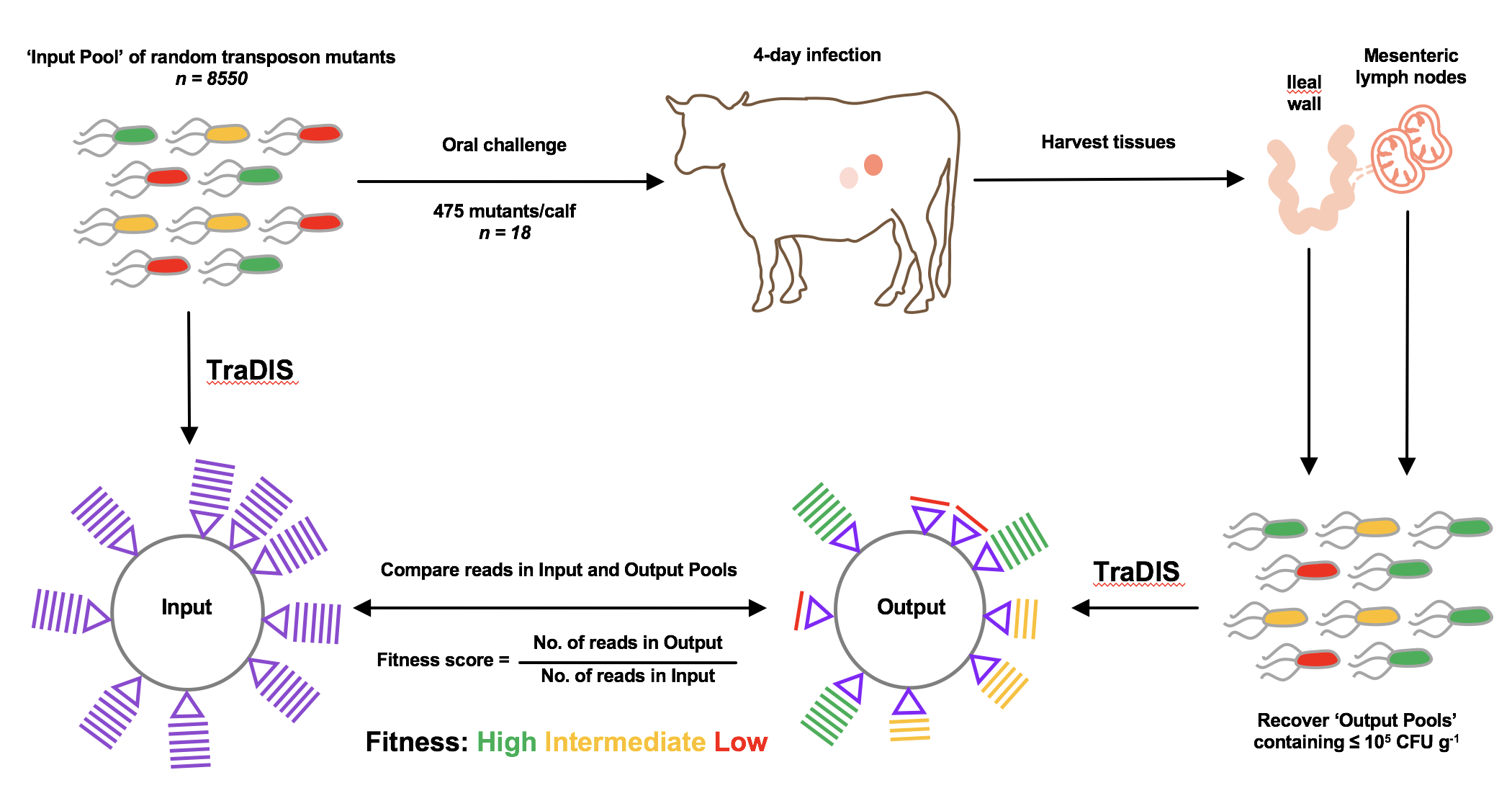

Supplement: Supplementary file 1 — Figure S1. Experimental strategy to screen TraDIS mutants in vivo. An input pool of random transposon-insertion mutants was generated and used to orally inoculate 28-day-old calves. Output pools of bacteria were recovered from tissues of interest, here the ileal wall and MLNs. Massively-parallel sequencing of the regions flanking each transposon allowed disrupted genes to be identified. A comparison of the number of sequence reads derived from the input and output pools at each transposon insertion allowed the relative fitness of each mutant to be assessed. Modified from Chaudhuri et al., 2013. (TIFF 386 kb) [file 12864_2018_5319_MOESM1_ESM.tiff]

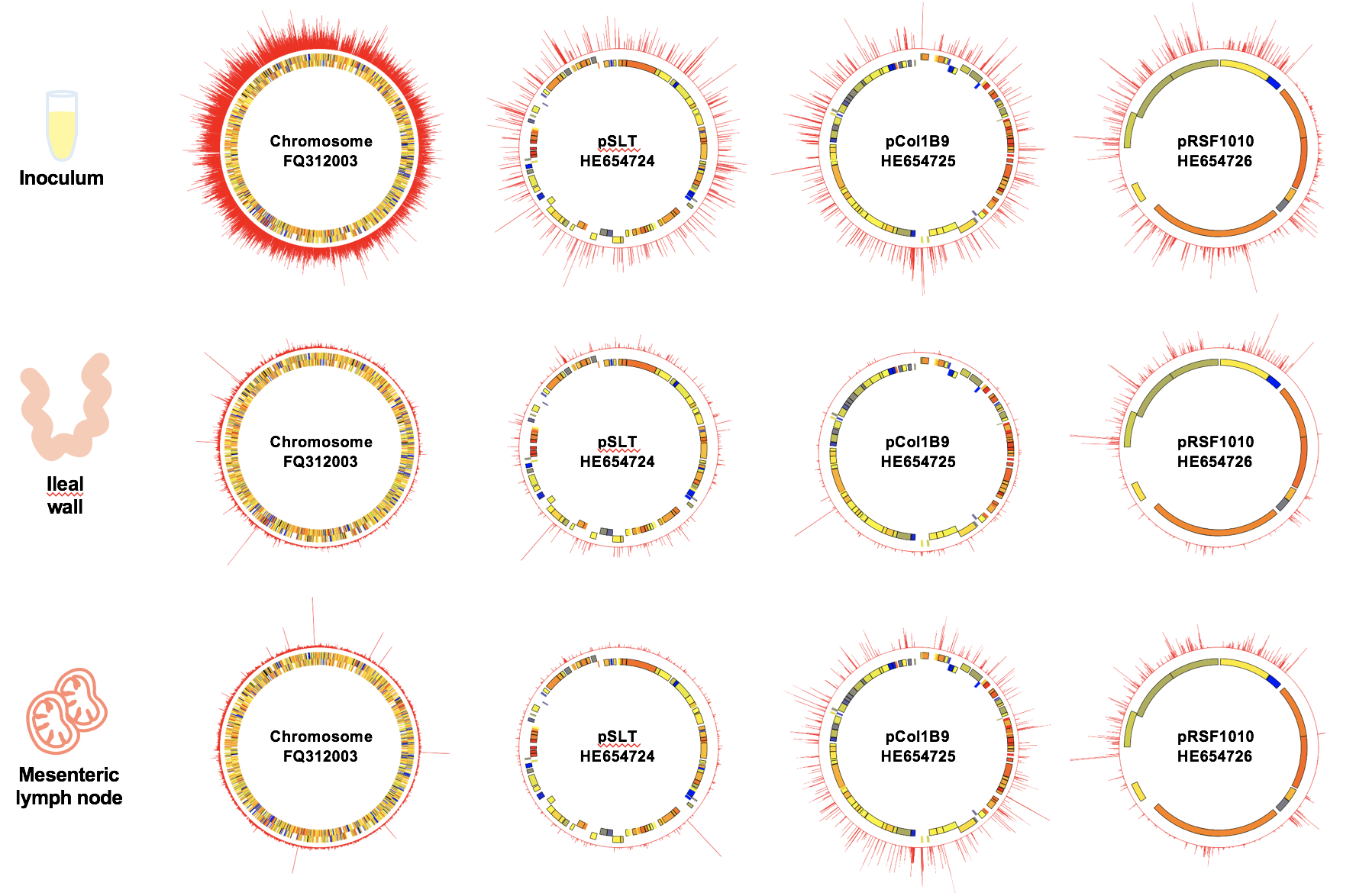

Supplement: Supplementary file 3 — Figure S2. Circular diagrams of the S. Typhimurium chromosome and plasmids showing the distribution and abundance of mapped transposon insertions. Transposon insertions were randomly distributed across the chromosome and plasmids of S. Typhimurium in the input pool and output pools from the ileal wall and MLNs. The inner two rings indicate the positions of annotated genes, coloured according to GC content (blue = low, yellow = intermediate, red = high). The outer ring indicates the number of transposon-flanking sequence reads obtained at each position. Peak heights are scaled relative to the highest peak in each diagram. (TIFF 914 kb) [file 12864_2018_5319_MOESM3_ESM.tiff]

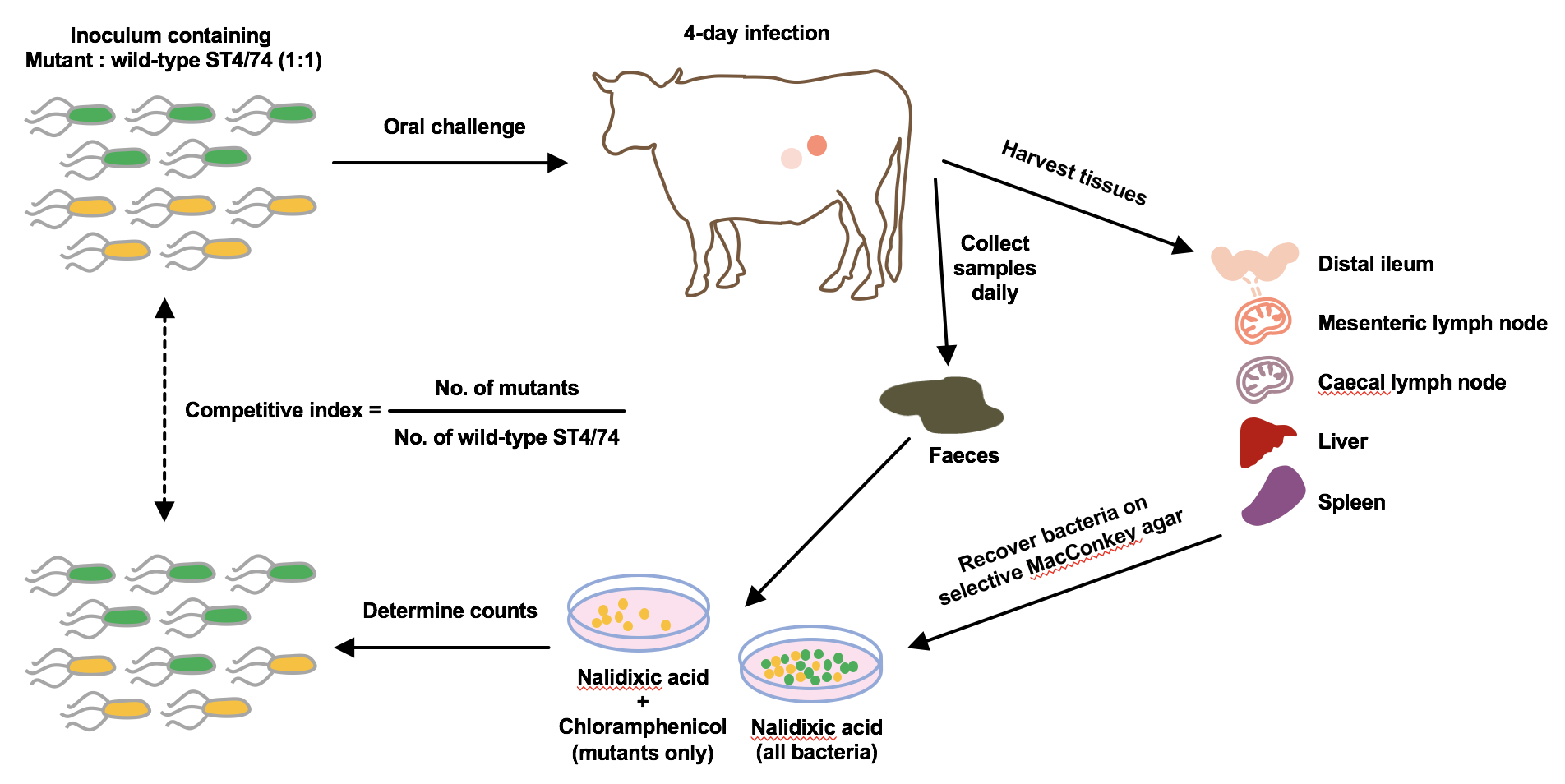

Supplement: Supplementary file 6 — Figure S3. Experimental strategy to validate attenuated mutants in vivo. To confirm the differential fitness of ptsN and spvC in the ileal wall and MLNs, null mutants were tested in competition with wild-type ST4/74 nalR in vivo. Calves were challenged orally with an inoculum containing mutant and wild-type bacteria in equal proportions. Bacteria were recovered from faeces daily and from the distal ileum, MLNs and other tissues at 4 days post-infection by plating on selective agar and the competitive indices were determined for each tissue. (TIFF 296 kb) [file 12864_2018_5319_MOESM6_ESM.tiff]
